# Post-appendectomy abdominal pain attributed to incidental ovarian cyst: a case report

**DOI:** 10.11604/pamj.2023.44.33.36316

**Published:** 2023-01-18

**Authors:** Konstantinos Ouranos, Aristarchos Almperis, Anastasia Kourti, Christos Kaselas, Ioannis Spyridakis

**Affiliations:** 1 4^th^ Medical Department, Hippokratio Hospital, Aristotle University of Thessaloniki, Thessaloniki, Greece,; 2 2^nd^ Department of Obstetrics and Gynaecology, Gynaecologic Oncology Unit, Aristotle University of Thessaloniki, Thessaloniki, Greece,; 3 2^nd^ Department of Pediatric Surgery, Aristotle University of Thessaloniki, “Papageorgiou” General Hospital, Thessaloniki, Greece

**Keywords:** Acute appendicitis, hemorrhagic ovarian cyst, progesterone, post-operative complications, case report

## Abstract

Acute abdominal pain in adolescents has a multitude of diagnoses to consider ranging from life-threating ones to other less obvious. In this case report, a 15-year-old girl presented with right lower quadrant abdominal pain and tenderness one month after successful surgical management of acute appendicitis. Post-appendectomy abdominal pain could easily be attributed to post-operative complications, while, in reality, a different disease state may be the cause of the pain. Physicians should have a high index of clinical suspicion, even though the temporal association of events may suggest otherwise. Hemorrhagic ovarian cyst (HOC) should be included in the differential, as it was confirmed with imaging in our case. A conservative treatment approach with progesterone was chosen, with menses resuming 2 days later, leading to regression of the cyst. The clinical significance of this case relies on the timely recognition of a disease entity, in order to distinguish it from complications arising postoperatively.

## Introduction

Acute abdominal pain is a common complaint in childhood and it poses a diagnostic uncertainty for clinicians, owing to the diversity of underlying surgical and non-surgical conditions. Acute appendicitis usually has a classic presentation, and hopefully, atypical manifestations are easily suspected, due to the high incidence of this surgical entity. However, the differential diagnosis of abdominal pain occurring after appendectomy is a major challenge for physicians, who should determine even uncommon conditions, apart from the most obvious ones, such as ileus and abscess [1]. In this case report, we present an unusual occurrence of a hemorrhagic ovarian cyst, manifesting after an unrelated bout of acute appendicitis.

## Patient and observation

**Patient information:** an otherwise healthy 15-year-old female visited the Emergency Department (ED) complaining of abdominal pain of two days duration localized in the right lower quadrant with progressing intensity. The pain was colicky in character, did not radiate, and was accompanied by a low fever of up to 37.5°C, nausea, and one episode of vomiting. The patient´s past medical and family history was unremarkable. She did not take any medications on a regular basis nor used tobacco, alcohol, or illicit drugs and had normal menstrual cycles since the age of 12 years old. Upon arrival at the ED, vital signs were normal. On physical examination, the abdomen was tender to palpation, especially in the right lower abdomen and the typical McBurney point was positive. The rest of the physical examination was unremarkable. Laboratory blood tests revealed leukocytosis with a left shift and increased C-reactive protein (CRP). Abdominal ultrasonography was performed that revealed a uterus and ovaries without pathologic findings and a small fluid collection within the lesser pelvic cavity. Right iliac fossa findings included an aperistaltic, non-compressible, dilated (>6 mm in diameter) and thick-walled mass, which was attributed to an edematous and inflamed appendix accompanied by hyperechoic peri-appendiceal fat and reactive iliac lymph node enlargement. The patient was admitted with the diagnosis of acute appendicitis, had intravenous fluid resuscitation, was started on antibiotics, and was taken to the theater for laparoscopic appendectomy. Intraoperative findings were unexpected. A pericaecal inflammatory mass (appendiceal plastron) that was firmly attached to the lesser pelvic cavity was identified. Laparoscopic mobilization of the bowel loops, in order to reveal the appendix, was not possible and was considered unsafe, so the operation was converted. An open approach was performed, bowel loops were released and the appendix was found to be gangrenous in consistency with a rupture due to the presence of a fecalith. Visualization of the adnexa was normal. Following appendectomy, purulent material was aspirated, the peritoneal cavity was washed with normal saline and the operation was completed. The post-operative period was uneventful and the patient was subsequently discharged from the hospital with no perioperative complications. One month after the operation, the patient returned to the hospital due to new-onset abdominal pain and multiple episodes of vomiting of one-day duration.

**Clinical findings:** vital signs were within normal limits and physical examination revealed moderate tenderness in the right iliac fossa without signs of peritoneal involvement.

**Timeline of the current episode:** October 2021: admission to the hospital for appendicitis and subsequent appendectomy. After 10 of hospitalization, discharge with follow-up instructions. November 2021: re-admission due to hemorrhagic ovarian cyst (HOC), conservative treatment, and discharge with follow-up instructions.

**Figure 1:**
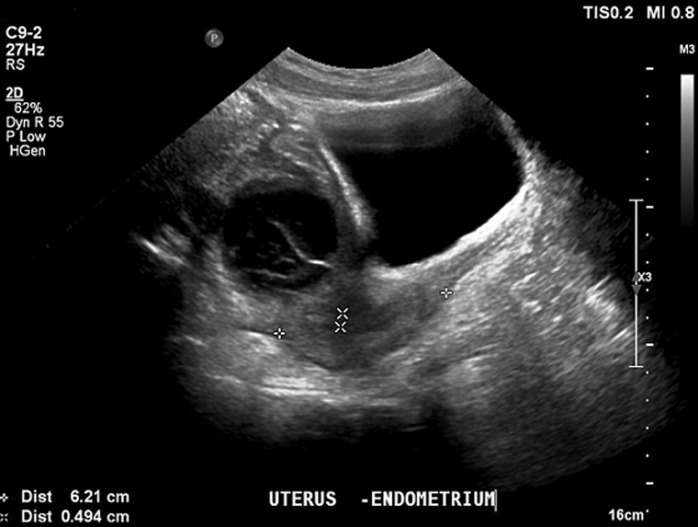
hemorrhagic ovarian cyst features on ultrasonography; ultrasonography revealing an ovoid cystic mass, with regular borders (47 x 45 x 56 mm), hyperechoic elements (possibly hemorrhagic features), multiple internal septations and absence of peripheral or internal vascularity

**Figure 2:**
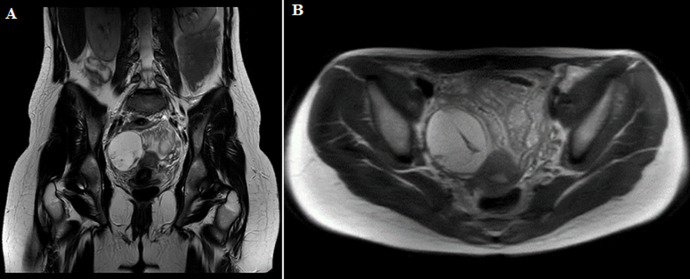
hemorrhagic ovarian cyst features on magnetic resonance imaging; coronal and axial pelvic MRI revealing an ovoid cystic mass, with internal septations and microhemorrhagic elements (6 x 5 x 6 cm)

**Diagnostic assessment:** laboratory test results were unremarkable. Due to the patient´s recent appendectomy, imaging was performed to rule out complications associated with the surgery, like intra-abdominal fluid collection or ileus. Ultrasonography (Figure 1) and computed tomography (CT) scan of the lower abdomen revealed the presence of a heterogeneous mass with a diameter of 6 cm in the right iliac fossa (RIF), a finding that could be attributed to an abscess, hemorrhagic ovarian cyst (HOC), or ovarian torsion. The suggested magnetic resonance imaging (MRI) of the pelvis confirmed the mass to be cystic in nature with internal septations and hemorrhagic elements and 6 cm x 5 cm x 6 cm in dimensions. Microcystic elements were also noted around the mass that could be attributed to ovarian follicles (Figure 2).

**Diagnosis:** the results of imaging examinations were compatible with HOC due to the presence of cystic mass with internal septations and hemorrhagic elements.

**Therapeutic interventions:** a more thorough patient history revealed that the patient had a 10-day delay in her usual menstrual cycle. Since her symptoms regressed and were not indicative of inflammation or acute surgical condition, and after obstetrician consultation, the mass was attributed to an ovarian hemorrhagic cyst and she was started on progesterone according to the patient´s age and weight. Two days later, she started menstruation, ultrasonography identified regression of the HOC, and the patient was discharged.

**Follow-up and outcome of interventions:** ultrasound of the lower abdomen on the fifth day of menstruation, which was unremarkable.

**Patient perspective:** when the patient was re-admitted she was concerned that she was coming across a possible post-operative complication. After the diagnostic work-up was completed and treatment was offered, she was fully relieved that her symptoms were due to menstruation abnormality.

**Informed consent:** it was sought and obtained from the patient. Anonymity was maintained for confidentiality.

## Discussion

Acute appendicitis is one of the most common surgical diseases in children with acute onset of abdominal pain. Pediatric surgeons should be familiar with recognizing not only the most typical symptoms but also evaluating accordingly the clinical presentation of a child, combined with a detailed history and the radiological findings, in order to avoid delayed diagnosis. In our case, the medical history and physical examination were suggestive of perforated appendicitis since the patient had been suffering from progressive and severe abdominal pain for 2 days prior to the ED visit, which was accompanied by abdominal rigidity [2-4].

Given the recent operation at the right iliac fossa, the reappearance of the patient with another episode of acute abdominal pain could mistakenly be attributed to a postoperative complication, such as an abscess, ileus, or fluid collection, and easily lead to misdiagnosis. Post-operative complications of appendectomy are typically infectious, with the most common being wound infection and intra-abdominal abscesses, and are more common in perforated appendicitis, like in our patient [4,5]. To evaluate for such outcomes, imaging studies were ordered and revealed a cystic ovarian lesion. This constellation of clinical and radiographic findings could point towards a diagnosis of ovarian cyst and/or ovarian torsion. The latter one was excluded, though, because the clinical presentation of this disease would be more severe and the ultrasonographic findings would show absence or reduced blood flow to the ovary. At last, taking into consideration the fact that the patient´s menses were delayed, the diagnosis of HOC was made.

Hemorrhagic ovarian cyst is a common cause of intraperitoneal hemorrhage in women of reproductive age, but it is uncommon in early adolescence. The presenting symptom in most cases is lower abdominal pain, owing to irritating qualities of the hemoperitoneum, which commonly has a sudden onset, among other symptoms, like nausea or vomiting [6]. A pregnancy test should always be performed and make sure to rule out an ectopic pregnancy. Ultrasonography is the preferred imaging modality for initial assessment, followed by a CT scan in case of ambiguous results, as it was performed in our patient. HOCs usually resolve spontaneously, and thus should be treated conservatively if there is no suspicion of massive intraperitoneal hemorrhage [6,7].

## Conclusion

Here, we presented a case of HOC, occurring shortly after appendectomy in a 15-year-old female. The typical clinical presentation of HOC may be difficult to differentiate from other causes, especially for pediatric surgeons, who are unfamiliar with this common gynecologic disease. Including HOC in the differential diagnosis of acute lower abdominal pain in young females is crucial, since its associated complications can be life-threatening. In the post-operative setting the clinician should always be in a position to first exclude possible post-operative complications but should always be vigilant of a new disease entity, such as HOC, that could be producing the new onset of complaints.
